# Evaluating Interactions of Forest Conservation Policies on Avoided Deforestation

**DOI:** 10.1371/journal.pone.0124910

**Published:** 2015-04-24

**Authors:** Juan Robalino, Catalina Sandoval, David N. Barton, Adriana Chacon, Alexander Pfaff

**Affiliations:** 1 Centro Agronómico Tropical de Investigación y Enseñanza, Turrialba, Costa Rica; 2 Universidad de Costa Rica, San Pedro, Costa Rica; 3 Norwegian Institute of Nature and Research, Oslo, Norway; 4 Sanford School of Public Policy, Duke University, Durham, North Carolina, United States of America; University of Waterloo, CANADA

## Abstract

We estimate the effects on deforestation that have resulted from policy interactions between parks and payments and between park buffers and payments in Costa Rica between 2000 and 2005. We show that the characteristics of the areas where protected and unprotected lands are located differ significantly. Additionally, we find that land characteristics of each of the policies and of the places where they interact also differ significantly. To adequately estimate the effects of the policies and their interactions, we use matching methods. Matching is implemented not only to define adequate control groups, as in previous research, but also to define those groups of locations under the influence of policies that are comparable to each other. We find that it is more effective to locate parks and payments away from each other, rather than in the same location or near each other. The high levels of enforcement inside both parks and lands with payments, and the presence of conservation spillovers that reduce deforestation near parks, significantly reduce the potential impact of combining these two policies.

## Introduction

Forest conservation policies are widely used strategies to preserve biodiversity and promote carbon sequestration around the world. Until recently, the evaluations of the effectiveness of conservation policies were scarce [1]. However, in recent years, they have become more popular and have been implemented in different countries (e.g. as noted in [2–16]).

Most of these evaluations consider forest conservation policies individually, when in fact different types of conservation instruments can be, and are, implemented jointly. The choices between different spatial arrangements and combination of policies could have important implications on their effect. However, the evidence of how policy effects change when policies are implemented simultaneously in one location is scarce [17], especially for forest conservation instruments [18], [19].

We evaluate the effects of policy interactions between two highly popular forest conservation policies: national parks and payments for ecosystem services. Forest conservation policies are not implemented randomly and, therefore, the different spatial configurations of policies, and the interactions generated, are likewise not randomly located. Simple comparisons of deforestation rates between areas with one policy or another, or the combination of both, and areas without any policy will likely lead to biased estimates of the causal effects. This is because important factors that drive deforestation can differ systematically between these areas.

We show that this is the case for Costa Rica. Lands inside parks without payments are significantly different from lands outside parks without payments. Similar results are obtained when we compare park buffers with unprotected lands. Moreover, this is also true for policy-mixes, which can be defined as “a combination of policy instruments which has evolved to influence the quantity and quality of biodiversity conservation and ecosystem service provision in public and private sectors” [18]. Parks with payments and payments in buffer zones are significantly different from unprotected lands. However, there are also differences between policies. Areas inside parks with and without payments differ significantly. Payments are located, on average, in more threatened lands than in the rest of the parks. There are also differences between payments inside parks, payments in park buffers and payments distant from parks.

One alternative to address this issue is to control for the differences in the characteristics of land between areas with different policy arrangements. To do that, we apply matching and regression methods. Within a policy mix (e.g. payments and protected areas), we search for observations similar to those with only payments, with only parks and without either policy. We then compare deforestation rates of these groups by running a regression to eliminate any remaining differences in land characteristics.

**Fig 1:**
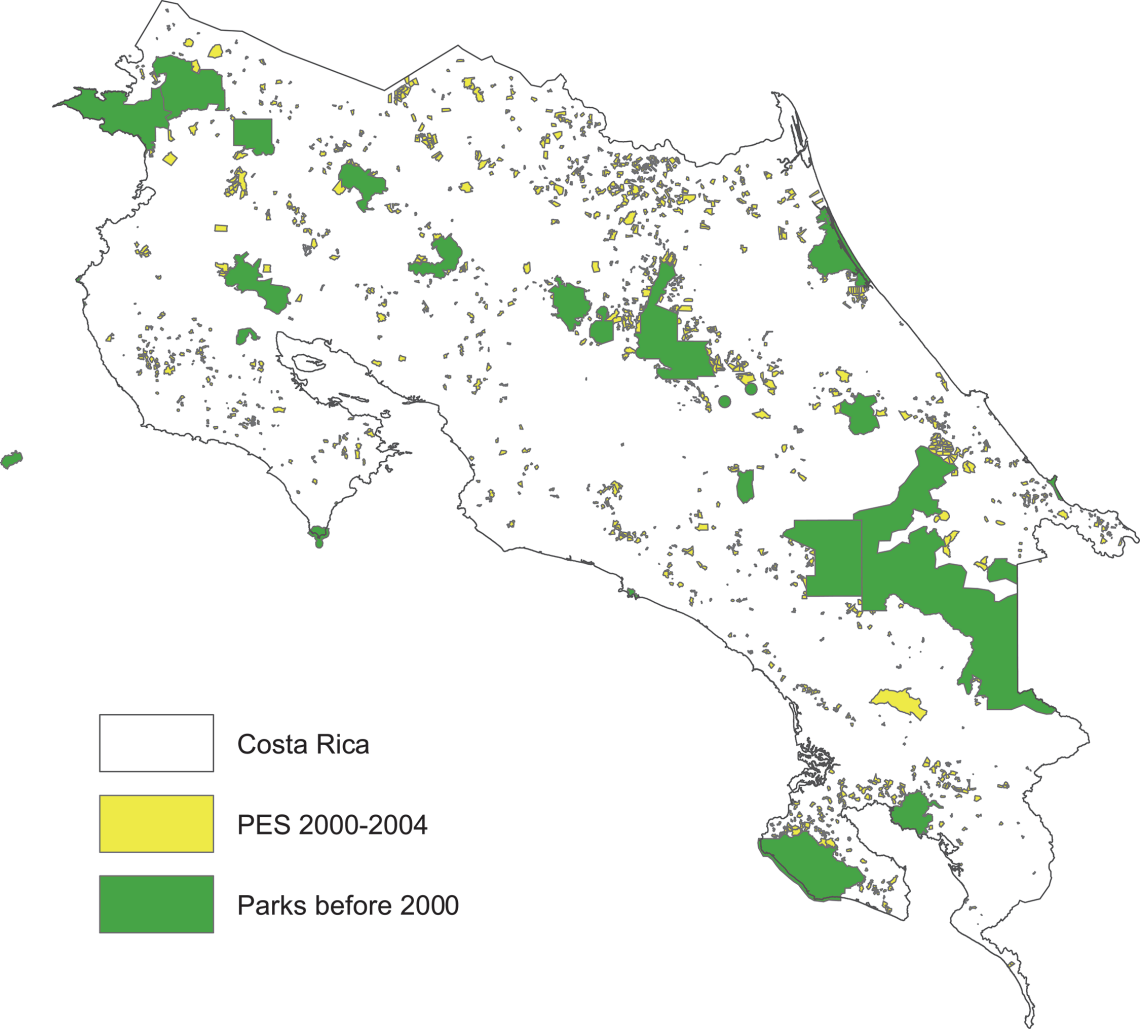
Protected areas and payments.

Costa Rica is an ideal place for this type of analysis. Forest conservation policies in Costa Rica are well supported and are spread all over the country (See Fig 1). National Parks cover 25% of the country and the payments program is a nationwide initiative from which landowners from all over the country have benefited. Additionally, both of these policies are relatively well enforced and monitored. Moreover, for the period 1963 to 1996, there is evidence that parks have reduced deforestation rates (see [6] and [7]) and that payments have reduced deforestation for the period 2000 to 2005 [20].

We find that the effects of implementing parks and payments separately are larger than implementing them together. The results were robust to different matching strategies. These differences are not statistically significant as there are few observations where these policies are combined. However, the magnitude of the difference is large, which suggests that the level of substitutability between these policies is high. This might be explained, in this case, by the fact that both of these policies are relatively well enforced in Costa Rica. Thus, once one is implemented the contribution of the other in the same location will be highly limited.

We also find that the effects of implementing payments distant from parks are larger than those implemented close to a park. The sum of the impacts generated by the payment distant from the park and by the forest conservation spillover effect from the park is higher than when the payment is implemented close to a park. Again, these results were robust to different matching strategies but statistically insignificant. It is important to emphasize, however, that this evidence applies only to the areas similar to where payments and park buffers were located. If other areas generate leakage instead of spillovers, these results could change.

## Effectiveness of Protected Areas (PAs) and Payments for Ecosystem Services (PES)

The effects of PAs on forest conservation have been widely studied (see [21] and [22] for overviews of the literature). Using regression and matching techniques, researchers have shown robust evidence that parks reduce deforestation in PAs compared to estimates of what would have occurred without the PAs (for instance, see evidence in [10] for Madagascar, [3] for Sumatra, [12] for the Brazilian Amazon, [9] for Panamá, [8] for Thailand, and [6] and [7] for Costa Rica). The tendency of PAs to be located in lower pressure areas does, however, lead to lower estimates of impact when using matching relative to only comparing deforestation rates between protected and unprotected lands [23].

The role that development plays on conservation effectiveness has also been well documented within this increasingly large body of evidence (see for instance [7], [24] and [25]). If they are well enforced, PAs’ forest impacts are higher when located in areas with higher threat of deforestation. One important implication of these results is that deforestation threat can be determined using easily observable indicators that could be used in defining new locations of PAs. For instance, there is evidence in Costa Rica and in Brazil that PAs located closer to cities and roads are significantly more effective in terms of reducing deforestation [7].

One concern with the implementation of PAs is that it requires buyouts to avoid deforestation produced by agricultural development, a fact that could affect living conditions [26]. One policy tool widely implemented to address this problem is payments for ecosystem services’ schemes, which compensate private landowners to maintain or increase the provision of ecosystem services [5], [27].

Whether this is an effective policy, in terms of additionality, has been widely discussed [28]. A large share of evaluation studies for PES has focused on Mexico and Costa Rica (see [29] for a review of rigorous evaluations), where countrywide programs have been established. Costa Rica’s PES program was one of the first payment programs in a developing country; it began in 1997. The forest law that created the program also significantly raised the hurdles for legal clearing of the forest in the entire country. Simultaneously, ecotourism activities increased and beef prices decreased. Attributing reductions of deforestation to the program was a challenge given all these changes. However, it was common to suggest that the program had a significant impact based on the observation that deforestation decreased and that enforcement was almost perfect as lands with payments remained in forest. Those facts, however, do not establish causation, which would require the proper estimation of counterfactual deforestation rates.

Using matching and regression methods that provide more accurate estimates of counterfactual deforestation rates, various analyses of the impacts of the PES program in Costa Rica were conducted. For the first years of the program, the estimates of impact were very low. There was no significant difference in deforestation rates between areas with and without payments [5], [30]. The estimates of the avoided deforestation generated by the PES program when restricting comparison to the most similar unpaid areas ranged from 0% to 0.20% per year of the land enrolled during the period 1997–2000 [15]. This implies that of every 500 paid parcels, only one would have been deforested per year and that was not due to the program. The matching analysis also showed that payments were located in lower than average deforestation threat areas. This suggests that the distribution strategy of the payments, which was on a first-come-first-serve basis, in addition to the presence of low deforestation due to the other factors mentioned, might have played an important role in limiting the impact of the program [15]. In addition, Mexico’s PES program shows statistically significant but, also, low impact on deforestation [13].

As in the case of protected areas, it is possible to target locations with higher clearing pressure [7], [24], and thereby raise the impact from PES. When analyzing the entire program between 2000 and 2005, the bias of payment towards low deforestation threat locations was reduced, and the impact increased [20]. For a similar period, [14] estimate even larger impacts in a region within Costa Rica known for an exemplary implementation and for the high levels of deforestation. Thus, it would be possible to increase PES impact by targeting higher threat areas, although such locations are likely to be more profitable in production and, as a consequence, have higher financial and political costs.

## Impacts of Policy Interactions

There are different ways in which policy combinations, and therefore, policy interactions, could take place [31]. For instance, interactions can take place between policies that have the same objective, e.g. parks and payments, or between policies that have different objectives, e.g. roads and parks [7], [24]. Interactions can also take place between policies at one governance level, e.g. local, or across levels, e.g. local and regional [31]. In our case, both parks and payments have the same objective and are implemented at the national level.

However, our main interest is the consequences of policy interactions according to their effects. Two important distinctions can be made. First, the interaction effects could be direct given that both policies intend to influence one group, as in the case of lands with payments and parks, or they could be indirect if the effects of at least one policy over the group are unintended, as in the case of lands in park buffers and payments. We will analyze these two types of interactions and the consequences on the effects on deforestation.

Second, policy interactions can also be classified into those that when combined reduce their effects, i.e. policy substitutes, or increase their effects, i.e. policy complements. More generally, interactions can be synergistic, complimentary, redundant, or conflicting [19]. Identifying how payments and parks and payments and park buffers interact in this respect is our main objective. Formally, for this paper, any pair of policies are substitutes if the reduction of deforestation caused by those policies, if implemented separately, is larger than the reduction of deforestation caused by those policies, if implemented in the same location.

One example of this is when land conservation policies with similar regulations are perfectly enforced. If a payment were implemented within a park, the additional effect on avoided deforestation would be significantly reduced, as the only additional gain from implementing the payment would be the payment conditions that are different from park regulations. If, for instance, both policies have the same regulation regarding deforestation, the additional impact of implementing a payment in a park will be null. In this case, the impact of implementing payments and parks separately will be significantly larger than when they are implemented together.

Substitutability could also occur between payments and park buffers. Ecotourism activities are heavily influenced by proximity to parks. This could generate reductions in deforestation rates [32]. If a payment is implemented in an area with low deforestation threat (close to a park) rather than in an area with high deforestation threat (far from a park) the impact of the payment on deforestation will decrease. When the reductions of deforestation due to the proximity to parks are large, is likely that the additional effect of the payment inside a buffer will be very low. Then, the reduction of deforestation caused by payments close to a park will be lower than if we consider the sum of the effect of payments away from parks and the effect of being close to a park. In Costa Rica, the lack of incremental effectiveness of PES in park buffers may also be explained by an increased presence of monitoring and enforcement as park officials patrol through buffer areas.

If enforcement is not strong, instrument complementarity may also be possible. Forest conservation policies could multiply the effects if implemented together. Parks regulate the possibilities landowners have in making use of their land resources. In some cases, landowners might be forced to violate these regulations as, for many of them, it might be their only source of income. If payments for environmental services are also available conditioned upon following additional regulations, beneficiaries might be more likely to comply with the regulations of both policies. This result would imply that the effects of these policies combined would be larger than the sum of the effects of these policies if implemented separately.

It is also possible that parks might be complemented by payments, but payments might not be complemented by parks. If, for instance, payments with perfect enforcement are given to landowners in parks with a low enforcement level, payments might increase compliance with park regulations. Then, the impact of parks will increase due to the presence of the payment. In this case, the park impact is complemented by the presence of the payment. However, the impact of the payment will not change with the presence of the park, as payments’ regulations will be well enforced inside or outside parks. Empirically, however, we will be able to estimate only the net effect of these relationships.

## Data and Methods

### Data

This paper is based only on information generated by public data using GIS. There was no fieldwork. Since there was none, this study did not involve any species, including those that are endangered or protected. We randomly selected 50,000 locations across a map of Costa Rica. Those are our units of observation. Our final sample of analysis has 14,510 locations. We excluded plots that were not forested by 2000 to study deforestation. In addition, to focus solely on forest conservation PES and on one of the various forms of protected areas, the National Parks, we also drop locations in any other form of protection, which include indigenous reserves, natural reserves, refuges, wetlands, forestry reserves and protected zones. Locations under PES contracts that were not of the forest protection type and locations in PES contracts without information on the category of protection were also dropped. We also eliminated locations in government parcels.

Our dependent variable is deforestation between 2000 and 2005. For a location, we observe land cover change in this period. Thus, if any location was covered by forest in 2000 but not in 2005, it is considered to have been deforested and is assigned a value of 1. If a location was still covered by forest in 2005, it is assigned a value of 0.

Concerning participation in PES, from FONAFIFO we received information about all of the farms that were engaged in the PES program in each year of the period 2000–2004. Specifically, we are focused on the contracts for Forest Protection, i.e., not those for reforestation or for sustainable forest management. Note that not all the area of a participating farm is necessarily participating in the program. Thus, even if PES is perfectly enforced (which seems an essentially correct assumption), it is possible to find deforested pixels inside farms that have some land enrolled in the PES program, due for example to within-property leakage effects. We excluded them: they are few in number and their inclusion did not tangibly affect the assessment of overall PES impacts [15].

We are able to identify locations inside parks and those that have both parks and payments. Most of the land inside parks is owned by the government. However, private lands remain inside parks. Given land use restrictions inside parks, when payments are distributed by the government agency, private lands inside parks are a priority.

**Fig 2:**
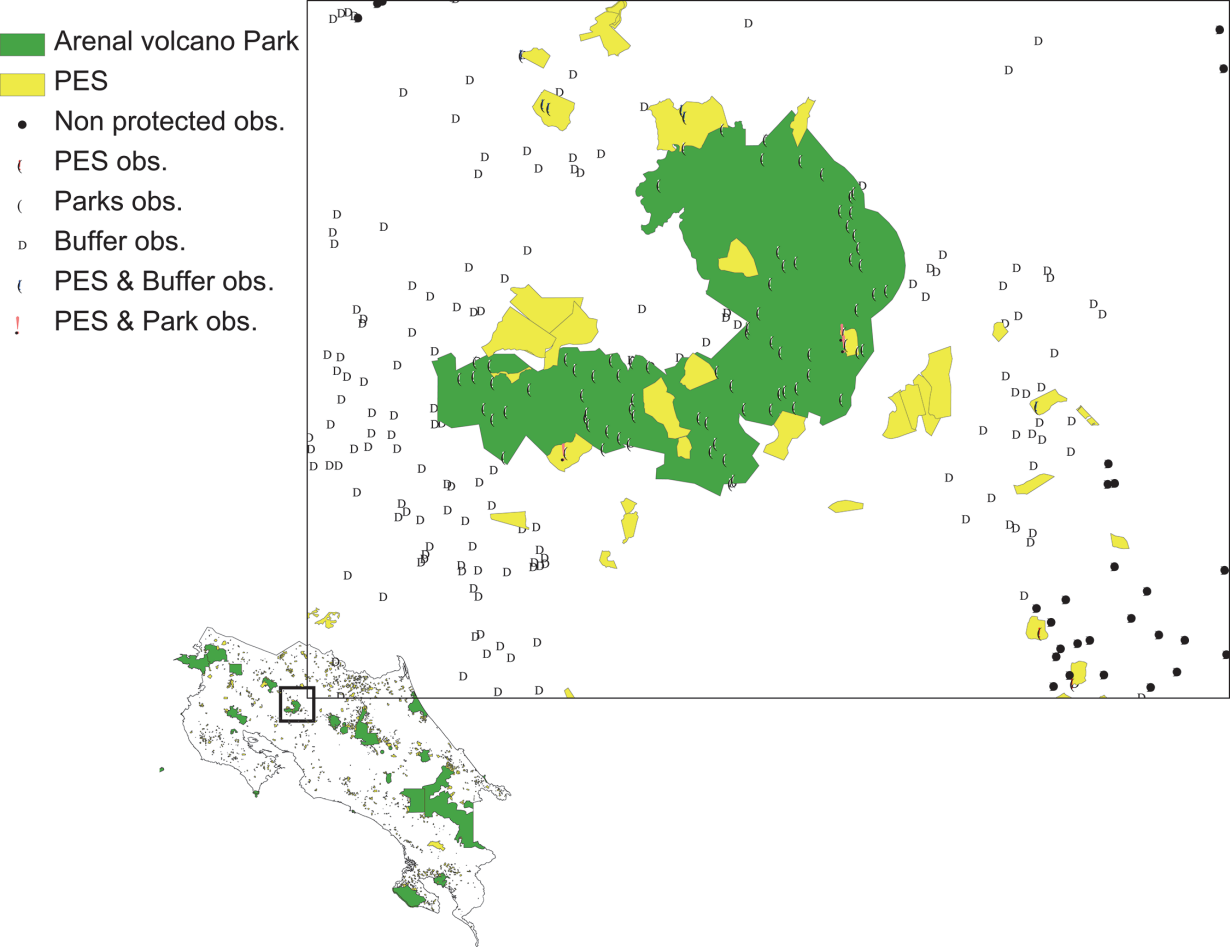
Description of treated and untreated observations.

We are also able to identify locations within 10km of national parks and, thus, also those observations receiving payments within 10km of a national park. Finally, we are also able to define untreated observations as locations that are at least 10km away from parks and that do not have payments. In Fig 2, we show some examples of locations and their classification within each of these groups.

We also have geographical characteristics of the locations such as precipitation, slope, and elevation, in addition to information about distance to local and national roads, distance to markets and ports and distance to the forest frontier. Finally, we also use information about life zones (based on Holdrich life-zone criteria) to create good, medium, and bad categories in terms of suitability for agriculture. In Table 1, we present descriptive statistics of all the variables used in the analysis.

**Table 1:** Descriptive statistics of treated and untreated observations by distance^1^ to Parks.

		Parks		Outside Parks			
				Buffer zone (0–10 km)		Outside buffer zone (more than 10 km)	
Variable		Without PES (1)	PES (2)	Without PES (3)	PES (4)	Without PES (5)	PES (6)
Deforestation rate (%)	mean	0.00	0.00	0.02	0.00	2.88	0.00
	se	0.00	0.00	0.00	0.00	0.00	0.00
Distance to forest frontier (m)	mean	2,288	877	239	363	171	262.84
	se	33	91	6	29	3	14.25
Distance to local roads (m)	mean	11,004	4,608	2,619	3,709	2,201	2,852
	se	93	477	48	166	26	127
Distance to national roads (m)	mean	15,192	6,654	3,898	5,176	3,635	5231
	se	139	560	67	263	44	211
Slope (grades)	mean	112	80	60	58	52	39
	se	1.63	13.06	1.72	5.91	1.11	4
Distance to San José (m)	mean	115,620	81,539	2,619	95,407	111,698	104,179
	se	736	5,378	48	3,307	615	2,491
Distance to Caldera (m)	mean	151,132	104,652	123,449	123,912	103,187	106,937
	se	675	5,789	991	3,134	718	2,658
Distance to Limón (m)	mean	126,772	133,784	169,346	132,035	178,712	166,323
	se	1,182	8,874	1,557	4,979	978	3,890
Distance to rivers (m)	mean	2,782	1,423	1,458	1,608	1,445	1461.12
	se	35	180	22	84	17	68.77
Life Zone Good (%)	mean	11.10	5.66	26.60	11.50	42.22	24.85
	se	0.00	0.03	0.01	0.02	0.01	0.02
Life Zone Medium (%)	mean	3.54	1.89	26.03	19.91	24.45	20.91
	se	0.00	0.02	0.01	0.03	0.01	0.02
Life Zone Bad (%)	mean	85.36	92.45	47.38	68.58	33.33	54.24
	se	0.01	0.04	0.01	0.03	0.01	0.03
Precipitation (mm)	mean	4,001	4,132	3,454	3,893	3,180	3,196
	se	16	118	19	65	12	53
Elevation (m)	mean	1286	1232	555	755	339	337
	se	13	103	11	47	5	20
# Observation		4740	53	2,974	226	6187	330

Note: se: standard error

1 Distance to National Park is in parenthesis.

### Empirical methodology

Empirically estimating how the impacts of policies change when implemented jointly is highly relevant but also rarely tested [17]. This is an issue that has been raised and addressed using randomized control trails in social sciences [33], [34]. Policy interactions are tested by using interventions (or treatments), and the combination of those interventions, randomly assigned [35]. Then, unbiased estimates of the effects of policies when implemented individually and together can be easily obtained by comparing treated and untreated outcomes. This is because, on average, each treated group and the control group, has, as expected, similar characteristics. Therefore, when comparing the outcomes of each treated group with the control group, we would obtain unbiased estimates. Moreover, when comparing treated groups, we would also obtain unbiased estimates of the difference of effectiveness between one policy or policy-mixes and the others. However, forest conservation policies are not implemented randomly and, therefore, their combination is not randomly located either. This creates two important empirical challenges. One is related to the unbiased estimation of the effects of each of the policies or policy-mixes. The other is related to the ability to compare effects of policies and policy mixes.

#### a. Unbiased estimates of policy impacts

In an empirical analysis, a simple mean comparison of deforestation outcomes between protected and unprotected areas would be a biased estimate of the impact because land differs not only in terms of protection status, but also in other important characteristics that determine deforestation. In that case, differences in means could not be attributed only to the protection. In fact, Table 1 shows that this also occurs in Costa Rica. Lands inside parks without payments (Column 1) are substantially different to lands outside parks without payments (Column 5). For instance, distance to forest frontier, distance to roads and slope are higher for locations inside parks than for locations outside parks. Similar results are obtained when we compare park buffers (Column 3) with unprotected lands (Column 5). This is also true for policy-mixes. Parks with payments (Column 2) and payments in buffer zones (Column 4) are significantly different than unprotected lands (Column 5).

To address this issue, we use propensity score matching (PSM) and multiple regression analysis. PSM consists in identifying similar untreated observations using observable land characteristics shown in Table 1 and comparing them with treated observations to remove the bias related to other observable explanatory variables [36]. The variables used in this analysis are those that reflect the likelihood of being deforested and protected as documented in [6], [7], [15]. The likelihood of being treated is used as a measure of similarity. Using the likelihood of being treated, each treated observation is matched to the n closest non-treated observations [37]. It is important to emphasize that the extent to which this methodology is effective in identifying causality effects depends on the availability of information. There is always the possibility that a cofounding factor is not observable and is therefore not included in the analysis. Then, we can find unprotected plots with a similar propensity score, which implies that they have similar observable characteristics to the treated observation. We, then, compare deforestation outcomes.

An issue requiring attention is the number of matched untreated observations that will be selected for every treated plot. Increasing the number of matches will give more variability; however that can also increase the bias [38]. In this case, we chose 20 and 30 matches and include a caliper that ensures that the untreated observations used are within 0.01 of the propensity score of each treated observation.

**Fig 3:**
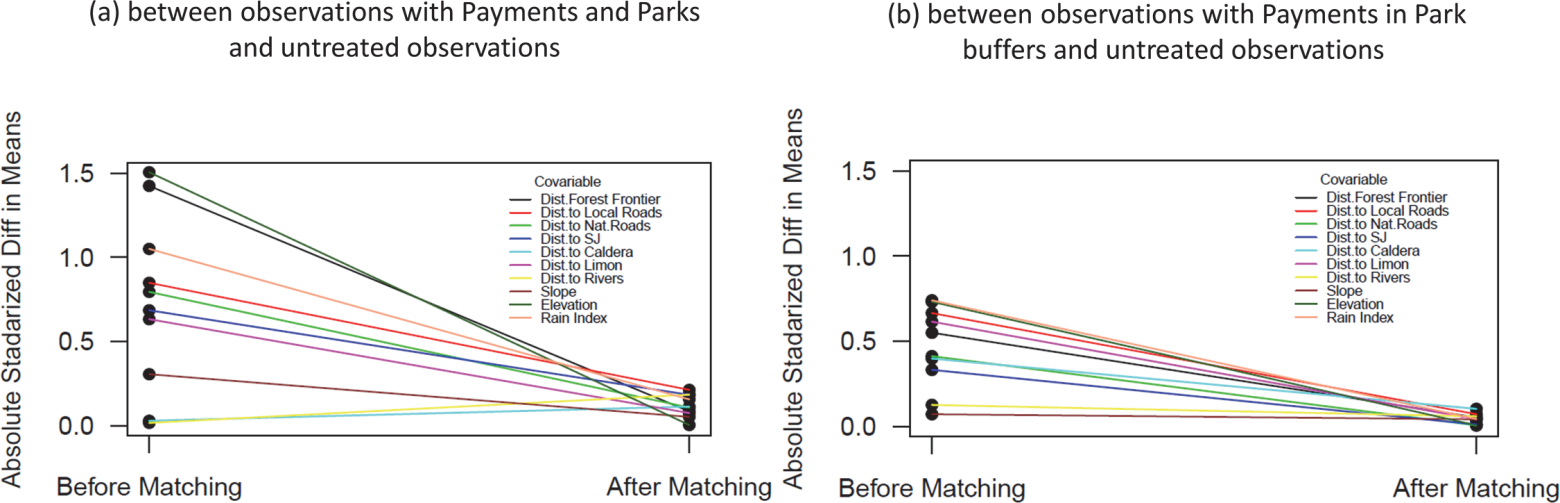
Absolute standardized differences before and after matching (n = 20, c = 0.01).

We then test if the comparison group found is similar to the treated group by looking at the differences in factors before and after matching. If we have a good comparison group, differences after matching should be lower than differences before matching. As a reference group for this analysis, we use observations that are both in parks and payments. In Fig 3, we show the difference before and after matching for n = 20 with a caliper of 0.01. We can observe the improvements in balances for most of the variables considered in the analysis after matching was conducted. The only variables in which the absolute standardized difference in means between control and treated observations increase are distance to Caldera (the Pacific port) and distance to rivers, but in both cases (before and after matching) the differences are statistically insignificant.

Under the matching approach the selection of matches is based on observable characteristics; however, there could be unobservable factors that drive deforestation and also the establishment of the protection policies. If there is a variable correlated with the implementation of the policy and also with the dependent variable that is not included in the regression, it would not be possible to find an unbiased effect of the policy on the outcome variable. This is an important caveat that applies, in general, to the use of matching and regression methods.

#### b. Adequate comparisons between policy impacts

However, our final goal is to adequately estimate the interactions generated by the combination of policies. To be able to estimate the interactions generated, we need to compare the effects of the policies when implemented separately from the effect of the policies when implemented jointly, as previously discussed. Comparing policies or treatment effects, when these are not randomly assigned, creates an additional challenge. This is because policy effects could significantly vary between one place of implementation and another. In fact, there is strong evidence showing that the impact of forest conservation policies is highly dependent on the characteristics that determine deforestation threat [7], [24], [25].

This implies that if we only compare the unbiased estimates of the impacts of policies at the national level, we would not know if the differences between the impacts of single policies and the combination of policies are due to the interactions between policies, or because policies were implemented in locations with different characteristics. For instance, it could be that the parks are implemented in low deforestation threat areas and payments are implemented in high deforestation threat areas. Then, the difference in impacts between parks and payments might be due solely to the difference in the location of these policies.

Similarly, in the case of policy mixes, it could be that policies and policy mixes are implemented in areas with different deforestation threat levels. If this is the case, when we compare the sum of the estimates of parks and payments implemented separately with the effects when implemented jointly, we would not know if the difference is due to the interactions between policies or due to the fact that they were implemented in different places.

In Table 1, we can see that this is the case in Costa Rica. There are significant differences in areas inside parks with (Column 2) and without payments (Column 1). Payments are located in more threatened lands that the rest of the parks. For example, parks with payments are nearer to forest frontier, roads and have less slope than parks without payments. However, payments outside parks and buffers (Column 6) are located in less threatened lands than unprotected areas (Column 5). There are also differences between payments inside parks and payments away from parks. Payments inside parks are farther from the forest frontier and national roads, for instance.

**Fig 4:**
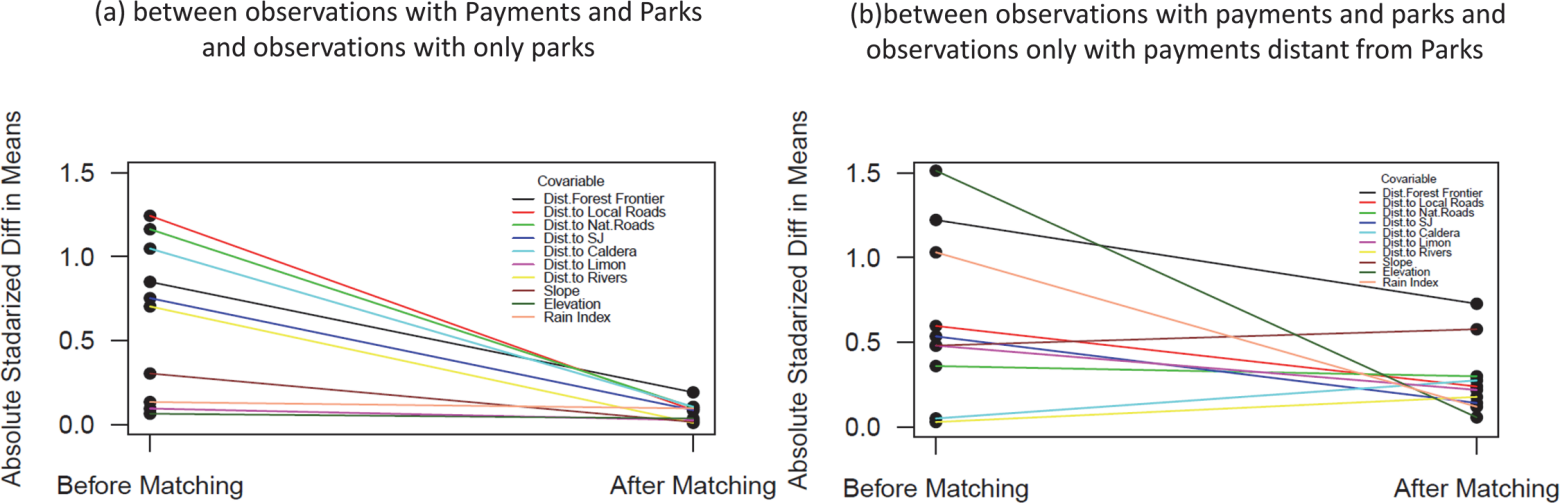
Absolute standardized differences before and after matching (n = 20, c = 0.01).

Matching is performed so that policy observations are also comparable. We make sure that observations from parks are similar to observations with payments inside parks. We also make sure that observations from payments away from parks are similar to observations with payments inside parks. Using matching techniques, we are able to obtain policy observations that are significantly more similar to each other (see Fig 4). We run the analysis only with matched policy and control observations.

## Results

In this section, we present the results of policy interactions. We divide the results in two sections. The first is a direct policy interaction analysis that focuses on the effects of payments being inside parks. The second is an indirect policy interaction analysis that focuses on the effects of being close to a park and inside payments.

### a. Parks and payments interactions

In Table 2, we present the results of estimating the impacts of various policy arrangements. The estimation of impacts of these policies is conditioned on the characteristics of lands with both parks and payments so that all those estimates can be compared. This also implies that all policy evaluations findings are valid only to groups with those characteristics. For those characteristics, we find that the impact of payments far from parks is statistically significant. The effects on reducing deforestation range from 1.15% to 1.61% (Column A). The impacts of parks implemented individually on reducing deforestation are also statistically significant, ranging from 0.9% to 1.23% (Column B). So, if we assume that policies are implemented separately in two plots, the sum of the effects on reducing deforestation will range between 2.05% and 2.85% of one forest plot (Column C).

**Table 2:** Policy impacts and interactions for observations similar to areas with payments and parks.

	A	B	C	D	E
Policy evaluated	*PES away from parks*	*Parks*	*PES away from Parks + Parks(A+D)*	*Parks and PES*	*Difference (D-C)*
PSM n = 20 cal. 1%	-0.0161**	-0.0123**	-0.0285***	-0.0125	0.0159
	[0.008]	[0.005]	[0.011]	[0.009]	[0.012]
PSM n = 20 cal. 2%	-0.0152**	-0.0120**	-0.0272***	-0.0122	0.0151
	[0.007]	[0.005]	[0.010]	[0.009]	[0.011]
PSM n = 30 cal. 1%	-0.0124*	-0.0096**	-0.0219***	-0.0097	0.0122
	[0.006]	[0.004]	[0.008]	[0.008]	[0.010]
PSM n = 30 cal. 2%	-0.0115*	-0.0090**	-0.0205**	-0.0091	0.0114
	[0.006]	[0.004]	[0.008]	[0.008]	[0.010]
Number of treated observations	330	4793		53	

Notes: PSM: Propensity Score Matching; cal.: caliper; n: number of matched controls observation. Standard errors in brackets.

*, **, *** indicates significance at 10%, 5% & 1%, respectively. We use distance to cities, distance to roads, distance to forest edge, distance to port, distance to rivers, distance to national parks, type of life zone, soil fertility index, rain index, elevation and slope as control variables in these regressions.

Now, we need to compare these results with what we would obtain if these policies were implemented in the same forest plot. In that case, we find that the effects on reducing deforestation range from 0.91% to 1.25%. However, these results are not statistically significant. Clearly, the effects of implementing both policies in two separate locations are larger than implementing the policy in only one. In Column E, we present the interaction that takes a positive value. This implies that once policies are jointly implemented the effect on reducing deforestation will be reduced, implying a relationship of substitution. This reduction ranges from 1.14% to 1.59%. However, the difference, even though large, is not statistically significant.

### b. Buffers and payments interactions

In Table 3, we present the results of estimating the impacts of various policy arrangements related to buffers and payments. The estimation of impacts of these policies is conditioned upon the characteristics of lands in park buffers with payments so that all those estimates can be compared. As before, this also implies that all policy evaluation findings are valid only for groups with those characteristics. For those characteristics, we find that the impact of payments far from parks is statistically significant. The effects on reducing deforestation range from 2.75% to 2.90% (Column A).

**Table 3:** Policy impacts and interactions for observations similar to areas with payments in buffers.

	A	B	C	D	E
Policy evaluated	*PES away Parks*	*Buffers*	*PES outside Parks+ Buffers(A+B)*	*Buffers with PES*	*Difference (D-C)*
PSM n = 20 cal. 1%	-0.0275***	-0.0152***	-0.0428***	-0.0281***	0.0147
	[0.009]	[0.005]	[0.011]	[0.010]	[0.014]
PSM n = 20 cal. 2%	-0.0284***	-0.0152***	-0.0436***	-0.0281***	0.0155
	[0.009]	[0.005]	[0.011]	[0.010]	[0.013]
PSM n = 30 cal. 1%	-0.0279***	-0.0153***	-0.0432***	-0.0275***	0.0156
	[0.009]	[0.004]	[0.011]	[0.010]	[0.014]
PSM n = 30 cal. 2%	-0.0290***	-0.0153***	-0.0443***	-0.0276***	0.0168
	[0.009]	[0.004]	[0.011]	[0.010]	[0.013]
Number of treated observations	556	3200		226	

Notes: PSM: Propensity Score Matching; cal.: caliper; n: number of matched controls observation. Standard errors in brackets,

*, **, *** indicates significance at 10%, 5% and 1%, respectively. We use distance to cities, distance to roads, distance to forest edge, distance to port, distance to rivers, distance to national parks, type of life zone, soil fertility index, rain index, elevation and slope as control variables in these regressions.

The differences between these impact estimates and the ones from Column A Table 2 are due to the fact that these two groups of payments away from parks have different characteristics. One group is similar to lands with parks and payments and the other is similar to lands in park buffers with payments. In Column 2 and 4 of Table 1, we show that there are important differences between these groups’ characteristics.

The average impact on reducing deforestation of buffer zones is statistically significant. The impact ranges from 1.52% to 1.53% (Column B of Table 3). So, if we assume that the buffer affects one plot and a payment another, the sum of the effects on reducing deforestation will range between 4.28% and 4.43% in terms of one forest plot (Column C of Table 3).

We again compare these results with what we would obtain if these policies were implemented in the same forest plot. In that case, we find that the effects on reducing deforestation range from 2.75% to 2.81%. These results are statistically significant. Clearly, the effects of having two plots, one in a park buffer without a payment and another plot with a payment outside the park buffer, are larger than implementing the payment in a park buffer. In Column E, we present the interaction that takes a positive value. This implies that once policies are jointly implemented the effect on reducing deforestation will be reduced and also that there is a relationship of substitution between the policies. This reduction ranges from 1.47% to 1.68%. However, the differences, even though large, are not statistically significant.

### c. Substitutability and complementarity

**Fig 5:**
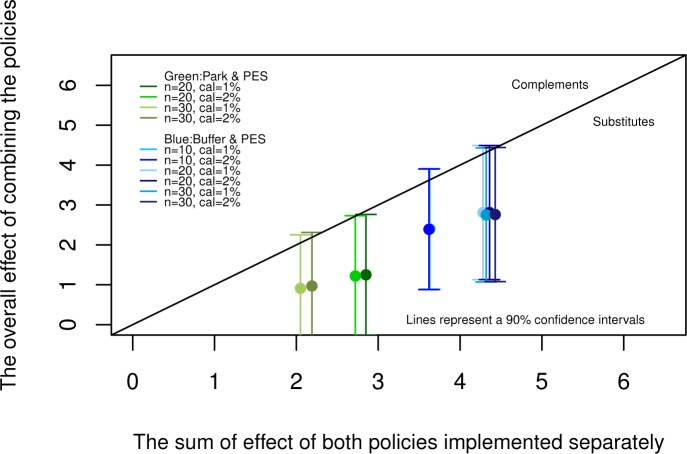
Interactions of policies.

In Fig 5, we describe whether policies are substitutes or complements. If the sum of the impacts of both policies implemented separately is higher than the overall impact of implementing both policies together, policies are substitutes (lower triangle). If the sum of the impacts of both policies implemented separately is lower than the overall impact of implementing both policies together, policies are complements (upper triangle).

In Fig 1, we show that there is robust evidence that the two forest conservation policies in Costa Rica have some degree of substitutability. All estimates are inside the lower triangle and away from the diagonal that represents the point where policies become complements. This occurs when we evaluate the interaction of the direct effects of payments and parks (green) as well as when we evaluate the interactions of the indirect effects though buffer zones and payments (blue). Confidence intervals, however, include the diagonal line.

## Conclusions

We evaluated the deforestation impacts that resulted from policy interactions between parks and payments and between park buffers and payments. To adequately estimate the effects of the policies and their interactions we use matching methods. Matching is implemented not only to define adequate control groups, as in previous literature, but also to define groups of locations under policy influence that are comparable to each other. We conducted this analysis in Costa Rica between 2000 and 2005. We found that it is more effective if one location is protected by a park and another by a payment than if one location is protected by both. Similarly, when analyzing park buffers and payments, we find that it is more effective to implement a payment outside the buffer zone than inside, because the buffer zone will already avoid deforestation without the payment. If the payment is located in a buffer zone, the total avoided deforestation will be lower than if that location is left only with the effect of the buffer and the payment is implemented in a location away from parks.

Enforcement might be playing an important role when analyzing direct interactions between parks and payments. Both policies, payments and parks are relatively well enforced. This implies that once one is implemented, it is unlikely that the other will have an important effect on reducing deforestation. In fact, in Table 2, it can be seen that Columns A and B intersect with Column D. This suggests that the implementation of the second policy in the same location is not generating any gain on avoided deforestation.

The sign of the effect on park buffers is also playing an important role in explaining the indirect policy interactions. We find that parks reduce deforestation in their neighboring areas (Table 3 Column B). This limits the effects that payments can have on avoiding deforestation. These results are valid only for areas that are similar to those where payments in buffer zones are located. The sign of these spillovers might vary according to land characteristics around protected areas. If the sign of spillover changes, the conclusions about the relationship between park buffers and payments might also change.

It is important to emphasize that the effects estimated for single policies are conditioned upon the characteristics of the land where policy mixes were implemented. With the objective of comparing policies, we use observations in each treatment status that had similar characteristics to where policy mixes were implemented. This implies that individual policy estimates of the effects only apply to areas with similar characteristics to where policy-mixes where implemented. This also implies that results could change if we focus on different land characteristics.

Finally, the variables used to eliminate the effects of confounding factors have been used in previous research with the same purpose (see for instance [6] and [7]). However, the possibility of a missing cofounding factor in the analysis still exists.
